# Functionalized Selenium Nanoparticles Synergizes With Metformin to Treat Breast Cancer Cells Through Regulation of Selenoproteins

**DOI:** 10.3389/fbioe.2021.758482

**Published:** 2021-10-04

**Authors:** Yu Yang, Zehang Zhang, Qi Chen, Yuanyuan You, Xiaoling Li, Tianfeng Chen

**Affiliations:** Department of Chemistry, and Institute of Food Safety and Nutrition, Jinan University, Guangzhou, China

**Keywords:** diabetes, concurrent tumor, combination therapy, nanometer pellet, oxidative stress, cell cycle arrest

## Abstract

Owing to high blood sugar level and chronic inflammation, diabetes tend to cause the overproduction of free radicals in body, which will damage tissue and cells, reduce autoimmunity, and greatly increase the incidence of tumors. Selenium nanoparticles (SeNPs) exhibit high antioxidant activity with anti-tumor ability. In addition, metformin is considered as a clinical drug commonly for the treatment of stage II diabetes. Therefore, in this study, different functionalized SeNPs combined with metformin were performed to detect the feasibility for cancer therapy. The combination of Tween 80 (TW80)-SeNPs and metformin was found to have a synergistic effect on MCF-7 cells. The mechanism of this synergistic effect involved in the induction of DNA damage by affecting the generation of reactive oxygen species through selenoproteins; the upregulation of DNA-damage-related proteins including p-ATM, p-ATR, and p38; the promotion of p21 expression; and the downregulation of cyclin-dependent kinases and cyclin-related proteins causing cell cycle arrest. Furthermore, the expression of AMPK was affected, which in turn to regulate the mitochondrial membrane potential to achieve the synergistic treatment effect.

## Introduction

Cancer, the second leading cause of death worldwide, is increasingly common among patients with chronic diseases such as diabetes (Yan et al., 2021). Diabetes is expected to become the seventh leading cause of death (Ratner, 2012). Oxidative stress, hyperglycemia, hyperinsulinemia, and chronic inflammation are hallmarks of diabetes and greatly increase the risk of cancer (Jalving et al., 2010; Abudawood, 2019). A higher tumor incidence has been reported in diabetes patients comparing with people without diabetes (Monami et al., 2011; Luo et al., 2020; Zhang et al., 2020; Park and Sandler, 2021). Breast cancer is among the cancers that are complicated by diabetes; its mortality rate is high in diabetes patients (Shelby et al., 2020; Park and Sandler, 2021). Chemotherapy, which is one of the main types of tumor treatment, has a comprehensive killing effect against tumors, (Jiang et al., 2019; Jabbarzadeh Kaboli et al., 2020; Xiang et al., 2020), but shows toxicity and side effects (You et al., 2016; Vojnits et al., 2019; D'Alterio et al., 2020). In addition, long-term use of chemotherapeutic medication can lead to multidrug resistance (Luo et al., 2019; Zhen et al., 2019). However, use of drug combinations in chemotherapy can reduce the toxic side effects of drugs and enhance their safety (Bang et al., 2019; Cai et al., 2020). The treatment of tumor patients with complex needs must take into account the interactions and contraindications among different diseases.

Metformin has the effect of lowering blood glucose, thereby reducing the incidence of tumors and inhibiting tumor growth. (Minett et al., 2012; Zi et al., 2015; Singh-Makkar et al., 2021). However, metformin alone requires a high dose for anticancer treatment, which is difficult to achieve safely in patients. Therefore, the therapeutic effect of metformin alone is poor. Many studies have considered the role of metformin as a sensitizer to chemotherapy, (Tan et al., 2019; Yang et al., 2020), radiotherapy, (Zannella et al., 2013; Bo et al., 2016), and immunotherapy (Xiong et al., 2021) in tumor treatment. Selenium nanoparticles (SeNPs) protect against anti-oxidative stress (Huang et al., 2020) and have anti-tumor effects (Liu et al., 2020; Ferro et al., 2021; Qi et al., 2021). They also show good biocompatibility, low toxicity, and antiviral, anti-tumor, and immunity-enhancing effects (Hu et al., 2019; Lai et al., 2019; Liu et al., 2019) and are thus promising for biological applications. However, the instability of SeNPs means that they are readily converted to inactive ash selenium or form aggregates, which reduces their bioavailability. The stability and cell absorption of SeNPs can be greatly enhanced by modification of polymers on their surface (Liu et al., 2016). For example, bovine serum albumin (BSA) has been used to modify SeNPs, resulting in BSA-SeNPs with enhanced stability and anti-tumor activity (Kong et al., 2011).

**SCHEME 1:**
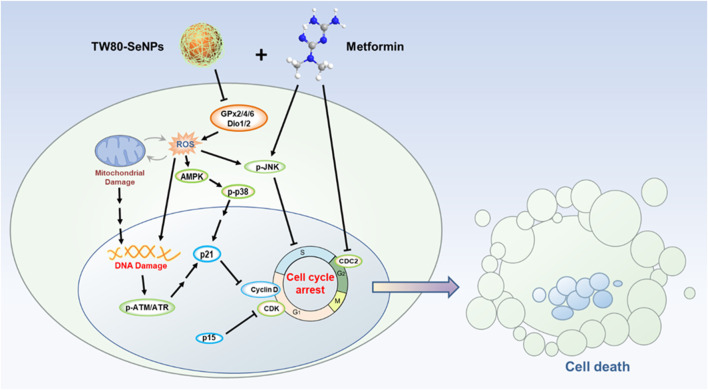
Schematic illustration of TW80-SeNPs combined with metformin to inhibit breast cancer cell proliferation.

Studies have reported that a combination of SeNPs with metformin shows a good synergistic effect in the treatment of diabetes (El-Rahim et al., 2017). Furthermore, this combination could alleviate pro-inflammatory cytokines expression and reactive oxygen species (ROS) production, thereby restoring antioxidant capacity (Liu et al., 2012; Chang et al., 2017). Therefore, we considered whether combining SeNPs with metformin for tumor treatment would result in a better anti-tumor effect, and whether the combination could represent a therapeutic option for patients with comorbid diabetes and tumors. In this study, we studied the use of functional SeNPs combined with metformin in the treatment of cancer, and conducted a simple study of the combination mode (Scheme 1). It was found that among different modified SeNPs, only TW80-SeNPs combined with metformin could inhibit tumor cell growth more effectively. A series of evidences showed that TW80-SeNPs could not only inhibit the expression of anti-oxidant selenoproteins and induced the production of ROS in tumor cells, thereby inducing mitochondrial dysfunction, but also could effectively up-regulate a variety of DNA damage-related proteins and cycle regulation-related proteins. These could synergize and enhance the cycle arrest effect of metformin on tumor cells, and ultimately slow down the progress of tumors by inhibiting the growth of tumor cells.

## Material and Methods

### Synthesis and Characterization of Selenium Nanoparticles Modified by Different Polymers

A 100 mM reserve solution of 0.5 ml Na_2_SeO_3_ was added to 1 ml of 20 mg/ml water reserve solution of PVP, PAH, or TW80, followed by addition of deionized water to a total of 8 ml with magnetic stirring for 5 min. Then, 2 ml of V_C_ 100 mM reserve solution was added into the mixed solution dropwise and stirred overnight at 25°C before being placed in a 10,000–20,000 kDa dialysis bag for 12–24 h.

Characterization of nano-selenium particles modified by different polymers: The hydration kinetic particle size and the zeta surface potential of the functionalized SeNPs were detected using a Malvin particle size analyzer, changes in the particle size were monitored in aqueous solution for a month, and changes in the particle size were monitored in PBS, DMEN with 10% FBS and human serum for 72 h. The morphology of the nanoparticles was observed by TEM.

### Cell Survival Assessment

Cell survival rates were detected by MTT assay: MDA-MB-231 cells and MCF-7 cells (in logarithmic growth phase) were inoculated in 96-well plates with a cell density of 2 × 10^4^ cells/mL. After cell adherent growth for 24 h, functionalized SeNPs (PAH-SeNPs, PVP-SeNPs, TW80-SeNPs) and exposed SeNPs (at concentrations of 80, 40, 20, 10, and 5 μM) and metformin (at concentrations of 50, 25, 12.5, 6.25, and 3.125 mM) were incubated for 72 h. After incubation, MTT (5 mg/ml, 30 μl/well) was added into each well, and all plates were placed in a 37°C incubator for 4–5 h. Then, the supernatant was discarded, dimethyl sulfoxide (150 μlL/well) was added into, and placed the plates in constant-temperature (37°C) shaker for 10 min to fully dissolve the blue and purple black formazan crystal. Finally, the 96-well plates were placed on a microplate analyzer to read the absorbance value (A) at 570 nm. The cell survival rate of each component was calculated: cell viability (% of control) = A_i_/A_0_ × 100%.

### Cell Colony Formation Assay

MCF-7 cells in logarithmic growth phase of 2 × 10^3^ cells/ml were inoculated into a 6-well plate. After cell adhesion growth for 24 h, TW80-SeNPs (5 μM or 10 μM) and metformin (2.5 mM or 5 mM) were added. The plate was placed in an incubator for 7 days, after which the medium was discarded, cells were washed with phosphate-buffered saline (PBS) for 3 times, and crystal violet was added for staining for 15–30 min. Then, the crystal violet was discarded and the cells were photographed.

### Cell Cycle Distribution Detected by Flow Cytometry

MCF-7 cells were placed in a 6 cm Petri dish with a density of 2 × 10^4^ cells/mL and treated with a predetermined drug concentration for 48 and 72 h; and the cells were washed with PBS and digested with trypsin. Cells were collected in a centrifuge tube and left to stand with the addition of 1–2 ml of 70% frozen ethanol. After centrifugation for 5 min and washing twice with PBS, and then resuspended. PI was added for staining at 25°C for 20–30 min in darkness. The cell cycle distribution was analyzed, selecting FSC, SSC, and PE as the channel parameters.

### Changes of Intracellular Reactive Oxygen Species

MCF-7 cells were digested with trypsin and resuspended after washing with PBS. Cell density was adjusted to 1.0 × 10^6^ cells/mL, and placed cells in a centrifuge tube in the dark. The appropriate amount of DCFH-DA probe was added and the tube was incubated for 30 min, shaking every 5 min to mix well. After centrifugation, the cells were resuspended with PBS and added to a 96-well plate containing control group, TW80-SeNPs (40 μM) group, metformin (10 mM) group, TW80-SeNPs (40 μM) combined with metformin (10 mM) group. Changes in fluorescence readings were continuously detected over 2 h using a BioTek Microplate system. Set the excitation wavelength to 488 nm and the emission wavelength to 525 nm. Taking the control group as reference, the percentage of the fluorescence intensity of the cells after drug treatment to the fluorescence intensity of the control group was calculated.

### Detection of Mitochondrial Membrane Potential (JC-1) Changes

In the JC-1 test, MCF-7 cells were inoculated into a 6-cm cell culture dish and allowed to grow on the wall for 24 h; then, TW80-SeNPs (40 μM) and metformin (20 mM) were added. After treatment with this combination for 4, 8, and 12 h, cells were digested with trypsin and collected, washed twice with PBS, centrifuged and resuspended. JC-1 dye probe was added, and the dye was placed in a constant-temperature incubator at 37°C for 30 min in the dark. During the dyeing process, the dye was shaken every 5 min to ensure that the dye and cells were fully mixed. Finally, a Beckman flow cytometer was used to detect the cell status, selecting FSC, SSC, FITC and PE channels as the channel parameters.

### Detection of Mitochondrial Morphology

In the mitochondrial morphology observation experiment, MCF-7 cells were inoculated into a 2 cm glass culture dish and allowed to grow on the wall for 24 h; then, TW80-SeNPs (40 μM) and metformin (20 mM) were added. The cells were treated with this combination for 2, 4, 8, and 12 h; then, a Mito-tracker red probe was added, followed by incubation for 2 h for intracellular mitochondria labeling. The cells were then incubated with Hoechst 33342 probe for 30 min to label the nuclei. The supernatant medium was discarded, 1–2 ml of PBS was added, and the cells were placed under a fluorescence microscope with a × 100 oil lens to obtain images.

### Statistical Analysis

All experiments were repeated at least three times. The results were expressed as mean ± standard deviation (mean ± SD). SPSS 13.0 was used for statistical analysis. The differences between the two groups of data were analyzed using the two-tailed *t* test. *p < 0.05, **p < 0.01, with significant statistical difference.

## Results and Discussion

### Synthesis and Characterization of Different Functionalized SeNPs

**FIGURE 1:**
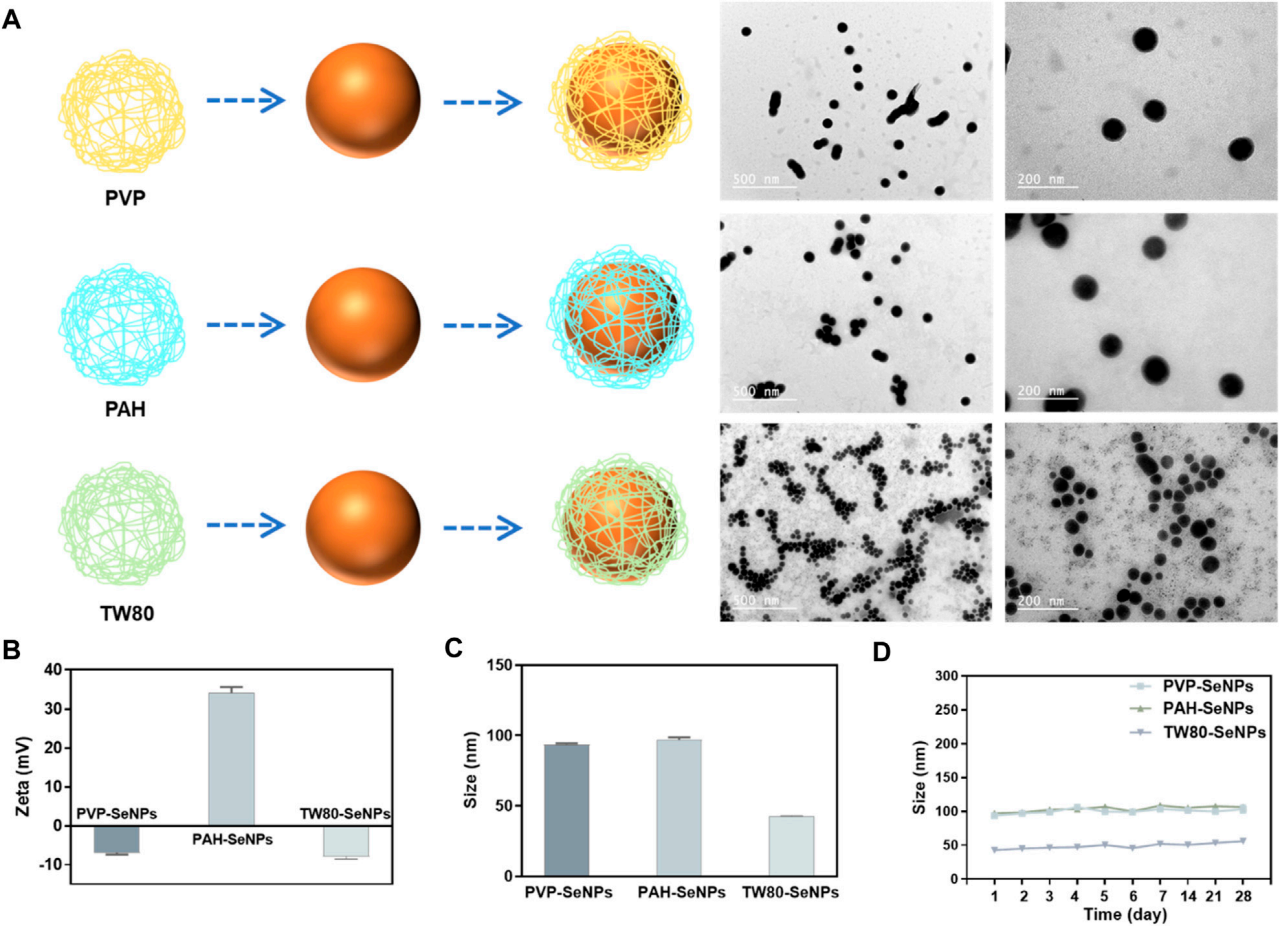
Synthesis and characterization of different functionalized SeNPs. **(A)** Synthesis and TEM images of functionalized SeNPs. **(B)** and **(C)** Zeta potential and size of different SeNPs. **(D)** Stability of different SeNPs in aqueous solution.

In order to synthesize different functionalized SeNPs with different surface modifiers, different positive and negative charges, and different sizes, we used polypropylene amine hydrochloride (PAH), polyvinyl pyrrolidone (PVP), and Tween 80 (TW80) to stabilize and modify SeNPs in a simple redox system (Na_2_SeO_3_+ Vitamin C) (Figure 1A). The synthesized SeNPs were confirmed to have different sizes and zeta potentials (Figures 1B,C). The PAH-SeNPs had a particle size of 100 nm and a high positive surface potential (approximately 38 mV). Transmission electron microscopy (TEM) images showed that they were uniformly distributed spherical nanoparticles (Figure 1A). The particle size of PVP-SeNPs was approximately 100 nm, and their surface potential was approximately −7 mV. TEM images showed that these nanoparticles underwent a small amount of agglomeration and their morphology was uniform. The TW80-SeNPs had the smallest particle size (50 nm) and a negative surface potential (approximately −8 mV). TEM images showed that their particle distribution was uniform and the particle size was small.

Evaluation of the physiological stability of nanoparticles is crucial to the development of their applications. Therefore, to verify the stability of the functionalized SeNPs, we monitored their particle size variation in aqueous solution over 1 month. As shown in Figure 1D, the functionalized SeNPs were stable for 1 month; after that, only a small amount of precipitate was observed in the PVP-SeNP solution. These results indicate that functionalized SeNPs can remain stable for a long time in aqueous solution. Furthermore, we have conducted the experiment to determine the stability of SeNPs in physiological conditions. As shown in Supplementary Figure S1, we found that the size of SeNPs was slightly decreased in DMEM with 10% FBS than PBS. Moreover, the size of the nanoparticles remained stable in human serum for 72 h, indicating the attenuated binding by serum proteins, and the high stability of the SeNPs under physiological conditions.

### Different Functionalized SeNPs Combined With Metformin Inhibited Proliferation of Breast Cancer Cells

**FIGURE 2:**
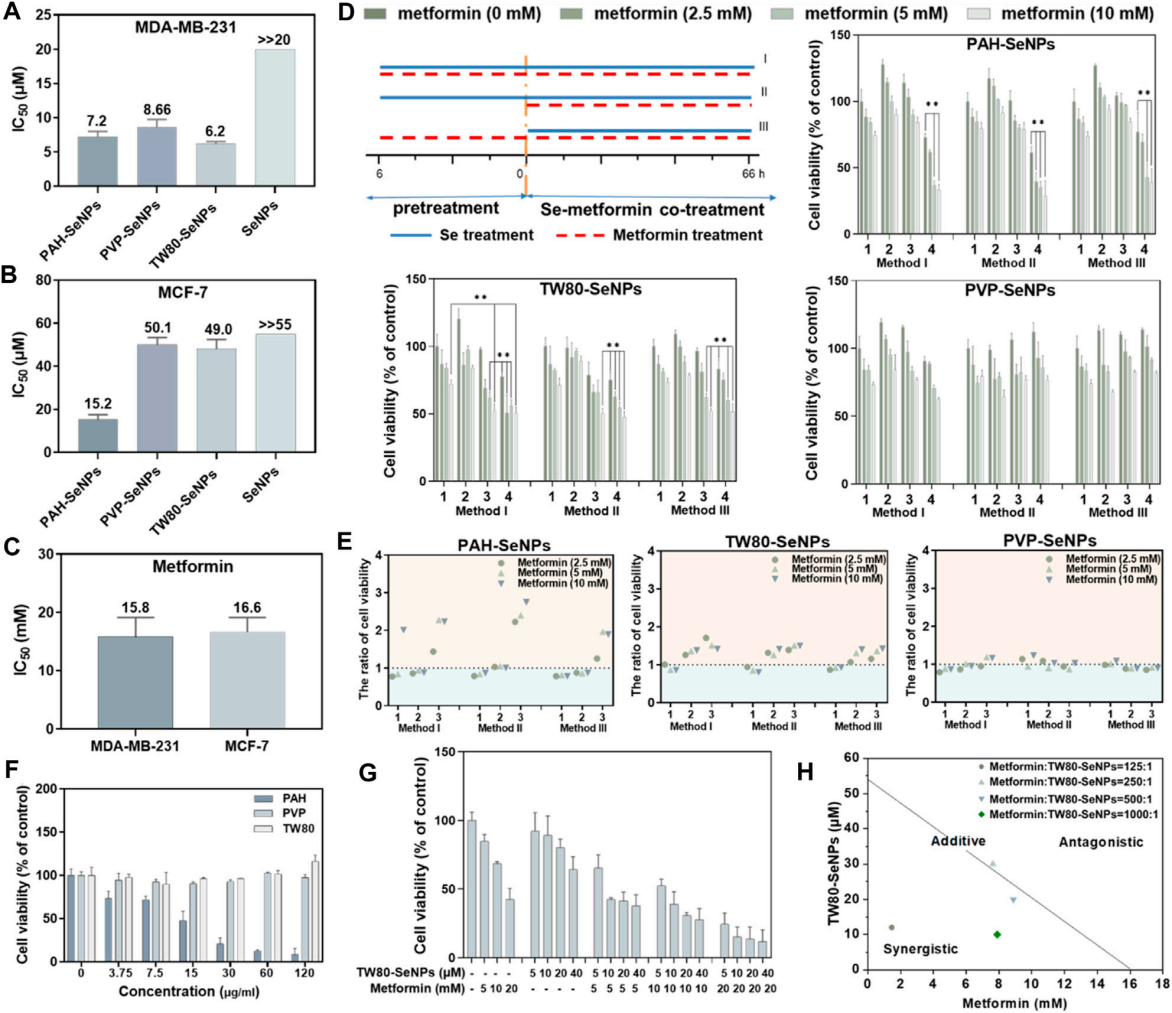
IC_50_ values of functionalized SeNPs to MDA-MB-231 **(A)** and MCF-7 **(B)** cells. **(C)** IC_50_ values of metformin to MDA-MB-231 and MCF-7 cells. **(D)** Effects on MCF-7 cell viability of different functionalized SeNPs combined with metformin under three treatment methods, where 1, 2, 3, and four represent Se concentrations of 0, 5, 10 and 20 μM, respectively. *p < 0.05, **p < 0.01, with significant statistical difference. **(E)** Survival rates of cells with and without SeNPs and the same concentration of metformin. The horizontal coordinates 1, 2 and 3 represent Se concentrations of 5, 10 and 20 μM, respectively. Points greater than one on the ordinate indicate some synergy. **(F)** Toxicity evaluation of PAH, TW80, and PVP with MCF-7 cells. **(G)** Evaluating the effects of TW80-SeNPs combined with metformin on MCF-7 cells. **(H)** Isobologram analysis of the antiproliferative effects of TW80-SeNPs and metformin on MCF-7 cells.

To verify the synergistic anticancer effects of functionalized SeNPs and metformin, we studied the survival rates of breast cancer cells (MDA-MB-231 cells and MCF-7 cells) treated with different functionalized SeNPs (PAH-SeNPs, PVP-SeNPs, and TW80-SeNPs) combined with metformin. As shown in Figures 2A,B, the half-maximal inhibitory concentration (IC_50_) of PAH-SeNPs in MDA-MB-231 cells was 7.2 μM, that of PVP-SeNPs was 8.66 μM, that of TW80-SeNPs was 6.2 μM, and that of the exposed SeNPs was much higher than 20 μM. In MCF-7 cells, the IC_50_ values of PAH-SeNPs, PVP-SeNPs, and TW80-SeNPs were 15.2, 50.1, and 49.0 μM, respectively, whereas that of exposed SeNPs was much greater than 55 μM. This was consistent with previous reports showing that exposed SeNPs had higher IC_50_ values and that functionalized SeNPs showed better anti-tumor effects. The IC_50_ values of metformin in 2 cells were 15.8 and 16.6 mM, respectively, as shown in Figure 2C. There was no difference in the anticancer effects of metformin between the two types of tumor cells. PAH-SeNPs showed good anti-tumor activity in MDA-MB-231 and MCF-7 cells, PVP-SeNPs and TW80-SeNPs showed strong killing effects against MDA-MB-231 cells, and metformin also showed a certain anti-tumor activity against both types of breast cancer cells.

Based on the IC_50_ values of the functionalized SeNPs in tumor cells, 20, 10, and 5 μM selenium were selected for a follow-up experimental study in MCF-7 cells. In MDA-MB-231 cells, 4, 2, and 1 μM selenium were selected for the follow-up experimental study. Concentrations of 10 mM, 5 and 2.5 mM metformin were also selected for subsequent experimental studies. To determine the combination with the optimal therapeutic effect, three different methods were used: (I) functional SeNPs and metformin co-incubated for 72 h; (II) functional SeNPs for 6 h, then functional SeNPs and metformin co-incubated for 66 h, and (III) metformin was used for 6 h and functionalized SeNPs and metformin co-incubated for 66 h.

As shown in Supplementary Figures S1–S3, the combination of TW80-SeNPs with metformin did not result in any significant enhancement compared with metformin alone or SeNPs alone of the anticancer effect against MDA-MB-231 cells under the three additive treatment modes. In the case of TW80-SeNPs, the anticancer effect was significantly enhanced when TW80-SeNPs (4 μM) and metformin were added at the same time, but there was no significant difference for the other methods and dose concentrations. For PVP-SeNPs, metformin (5 mM, 2.5 mM) significantly enhanced the anticancer effect against tumor cells after co-incubation with PVP-SeNPs (4 μM) for 6 h, but there was no significant difference with the other methods and dose concentrations. Therefore, the synergistic effects of PAH-SeNPs, TW80-SeNPs, and PVP-SeNPs combined with metformin were not obvious in MDA-MB-231 cells.

As shown in Figures 2D,E, in MCF-7 cells, PAH-SeNPs (20 μM) combined with metformin (10 mM or 5 mM) could kill tumor cells, and there were significant differences compared with metformin alone or SeNPs alone. TW80-SeNPs (20 μM or 10 μM) combined with metformin could kill MCF-7 cells, and the difference was significant compared with metformin alone or SeNPs alone. There was no significant difference among the three administration modes of PVP-SeNPs and metformin. Therefore, PAH-SeNPs and TW80-SeNPs combined with metformin may have a synergistic effect on MCF-7 cells.

To verify whether the enhanced effect of functionalized SeNPs combined with metformin was affected by surface modification of SeNPs, the toxicities of PAH, PVP, and TW80 alone to MCF-7 cells were detected, at concentrations corresponding to selenium concentrations of 80, 40, 20 , 10 and 5 μM. As shown in Figure 2F, the results of the MTT assay showed that PAH alone was toxic to MCF-7 cells, whereas PVP or TW80 alone had no toxic effect on MCF-7 cells. This suggested that the enhanced effect of PAH-SeNPs combined with metformin might be related to PAH. Based on the above results, a follow-up experimental study was carried out, in which TW80-SeNPs and metformin were co-incubated with MCF-7 cells. An enhanced effect of TW80-SeNPs combined with metformin on cells was again detected by MTT, and the isobologram method was used to analyze it. As shown in Figures 2G,H, the anticancer effect was significantly enhanced when TW80-SeNPs and metformin were added at the same time, and there was a synergistic effect when the ratio of metformin to TW80-SeNPs was 125:1, 500:1, or and 1,000:1; when the ratio was 250:1, there may have been a simple additive effect. In sum, TW80-SeNPs with a highest cytotoxicity was suitable for sensitizing metformin. Furthermore, the individual TW80 showed high biosafety in a concentration at 120 μg/ml. Thus, TW80-SeNPs and metformin have a synergistic effect in anti-tumor therapy against MCF-7 cells.

### Cellular Uptake of Functionalized SeNPs by MCF-7 Cells

**FIGURE 3:**
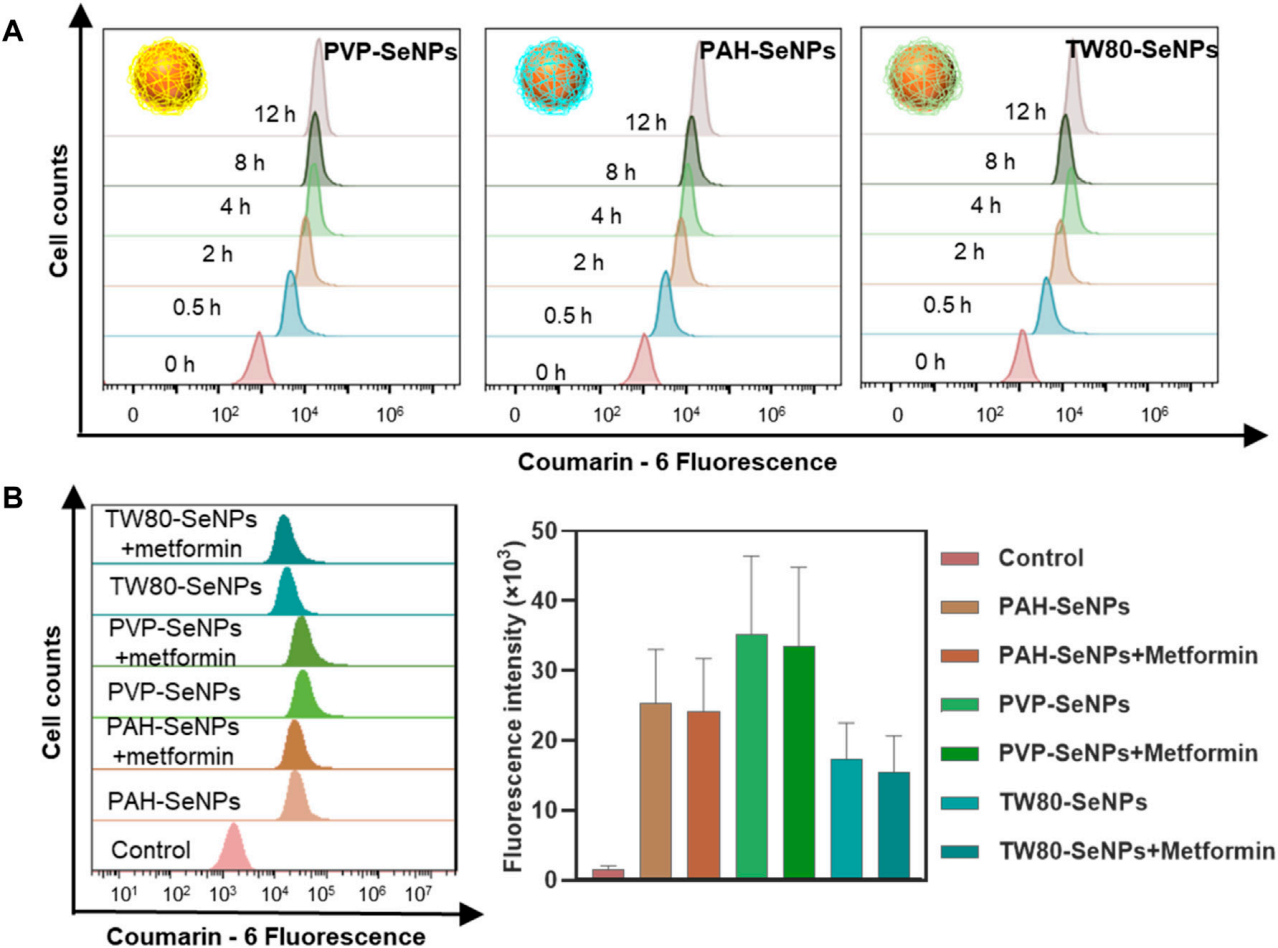
Cellular uptake of functionalized SeNPs by MCF-7 cells. **(A)** Cellular uptake of functionalized SeNPs in MCF-7 cells. **(B)** The flow cytometric results and statistical graphs of the cellular uptake of functionalized SeNPs in MCF-7 cells with the presence of metformin.

We next considered whether the differences in the enhanced effects of functional SeNPs combined with metformin on MCF-7 cells were due to the different amounts of different functionalized SeNPs absorbed by cells. We compared the absorption of functionalized SeNPs by MCF-7 cells at different time points. In Figure 3A, the absorption of selenium by MCF-7 cells was time dependent and reached its maximum at 4–8 h. At 8 h, there was no significant difference in absorption among the three types of functionalized SeNPs. To verify whether the absorption of selenium by cells was changed by the addition of metformin, we compared the absorption of SeNPs by cells in presence of metformin and absence of metformin. As shown in Figure 3B, the presence of metformin did not affect the absorption of any of the three types of SeNPs, and there were no significant differences in absorption among the three types. Therefore, the enhanced effect of the combination of drugs may not be due to differences in the absorption of SeNPs by cells. The specific sources of differences need to be further studied.

### Effects of TW80-SeNPs Combined With Metformin on Cell Cycle Distribution of MCF-7 Cells

**FIGURE 4:**
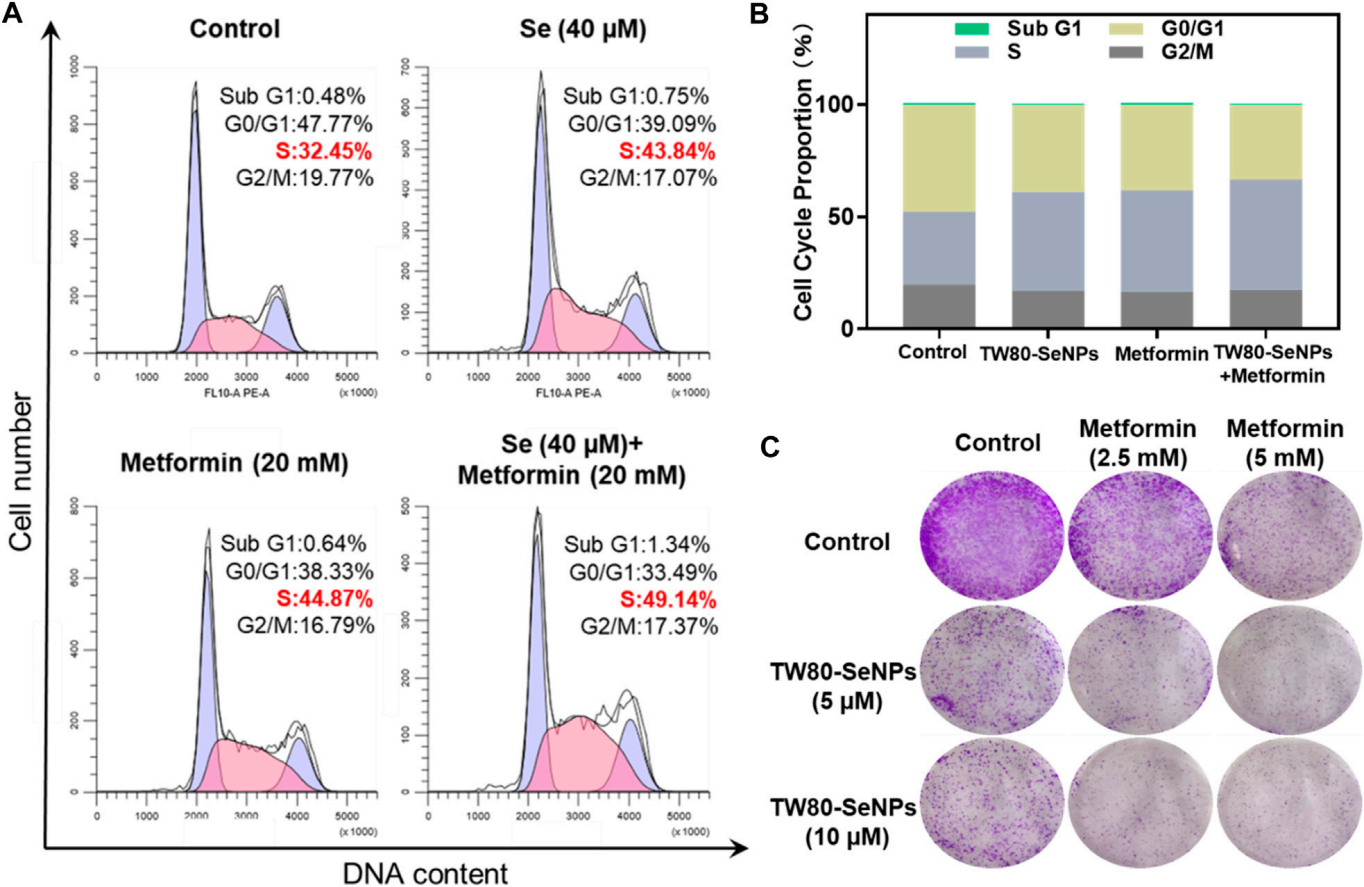
**(A**,**B)** Flow cytometric analysis of MCF-7 cells after treatment with TW80-SeNPs and metformin for 48 h. **(C)** Anti-invasion effects of the treatment. Effects of TW80-SeNPs, metformin and co-administration on colony formation of MCF-7 cells at 7 days.

The cell cycle plays an important part in the study of anti-tumor mechanisms (Huang et al., 2020). Here, flow cytometry was used to detect the effects of TW80-SeNPs combined with metformin on the cell cycle distribution of MCF-7 cells. As shown in Figures 4A,B, the proportion of S phase cells after treatment with TW80-SeNPs (40 μM) was 43.84%, compared with 44.87% after treatment with metformin (20 mM) and 49.14% after treatment with TW80-SeNPs combined with metformin. Compared with the blank control group (32.45%), the proportions of S phase cells in the TW80-SeNPs group and metformin group increased significantly, and the proportion of S phase cells increased more significantly after combined use of TW80-SeNPs and metformin. However, there was almost no significant change in the distribution of Sub-G1. Therefore, TW80-SeNPs combined with metformin can inhibit the growth of tumor cells by affecting cell cycle arrest, resulting in an enhanced anti-tumor effect.

The enhanced effect of TW80-SeNPs combined with metformin was further verified by colony formation assay. As shown in Figure 4C, the cells growth in the metformin group was significantly inhibited. The growth of cells in the TW80-SeNPs group was inhibited. The inhibitory effect was more obvious in the TW80-SeNPs combined with metformin group than in the TW80-SeNPs-alone or metformin-alone groups, that is, it was stronger with the combined treatment.

### Exploration of Oxidative Stress Mechanism in McF-7 Cells Under Treatment

**FIGURE 5:**
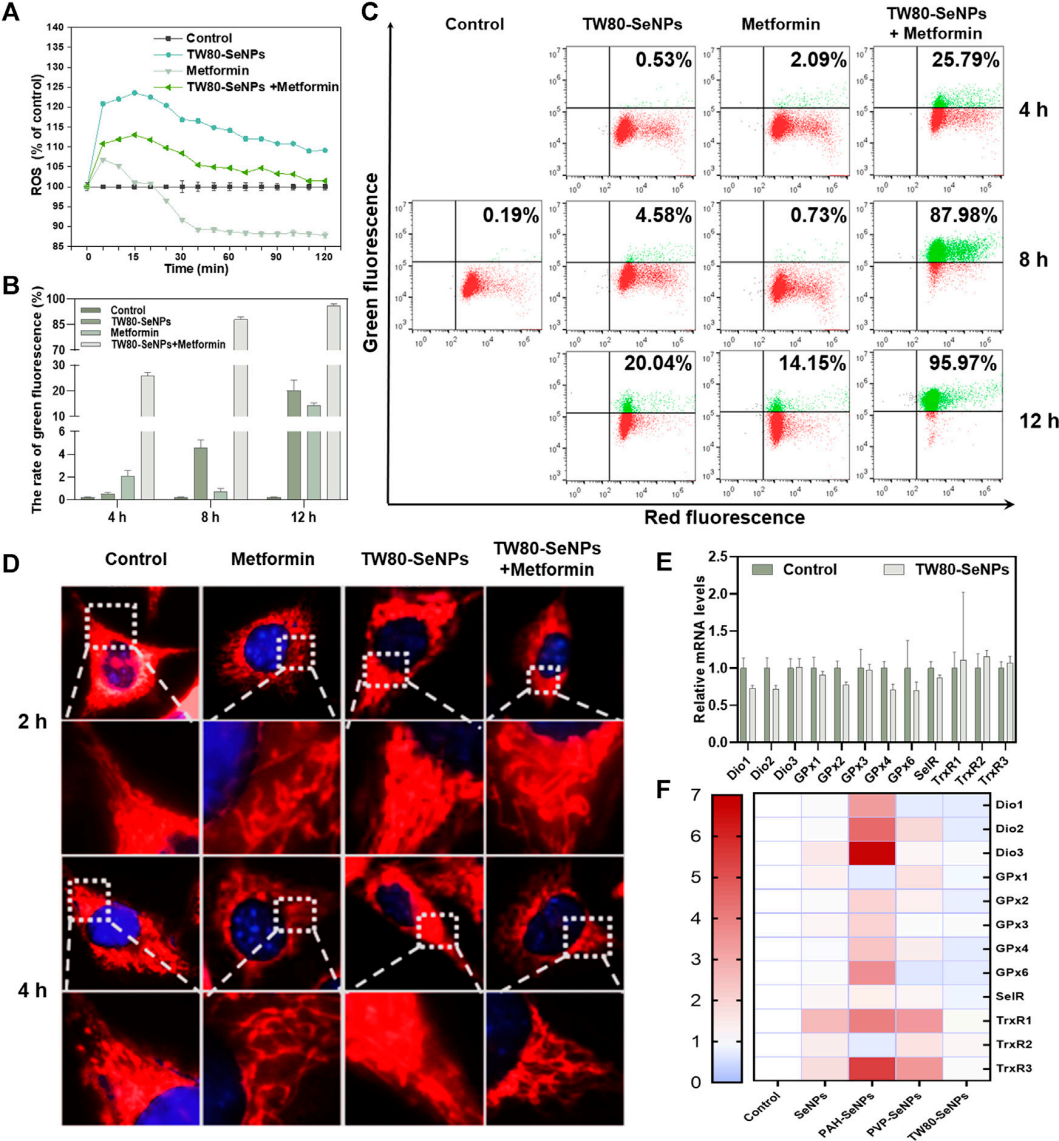
Changes of ROS levels, mitochondrial morphology, mitochondrial membrane potential and expression levels of selenoproteins in MCF-7 cells. **(A)** ROS levels in MCF-7 cells treated with TW80-SeNPs (40 μM) and metformin (10 mM). **(B)** and **(C)** Mitochondrial membrane potential of MCF-7 cells treated with TW80-SeNPs (40 μM) and metformin (20 mM) at different time points. **(D)** Mitochondrial morphology and representative magnified images of MCF-7 cells treated with TW80-SeNPs (40 μM), metformin (20 mM) and co-administration at different time points. **(E)** and **(F)** Expression levels of selenoproteins in MCF-7 cells after different treatments.

Levels of intracellular ROS can affect the growth and proliferation of cells (Wang et al., 2020; You et al., 2020). Excessive production of ROS causes oxidative stress in cells and subsequent DNA damage, (Indo et al., 2007) this results in changes in cell cycle distribution and thus affects cell lifespan (Ratner, 2012). ROS play the important role in the killing of tumor cells by SeNPs, as has been widely reported (Guo et al., 2017; Soumya et al., 2018; Rao et al., 2019). Thus, we detected levels of ROS in MCF-7 cells treated with TW80-SeNPs, metformin, or the combined treatment, respectively. As shown in Figure 5A, the TW80-SeNPs group showed a significant increase in intracellular ROS levels compared with the control. In the first 15 min, metformin increased intracellular ROS levels and then decreased them. In the combined treatment group, the intracellular ROS levels also significantly increased, but slightly less than in the TW80-SeNPs group; however, this did not affect the enhanced effect of the combined treatment. These results indicate that an increase in ROS levels may be an important factor in the enhanced anti-tumor effect of the combination of the two drugs.

Excessive production of ROS can cause mitochondrial damage and affect mitochondrial membrane potential (Ma et al., 2020). Therefore, changes in mitochondrial membrane potential of MCF-7 cells were detected at 4, 8 and 12 h. As shown in Figures 5B,C, neither metformin alone nor TW80-SeNPs alone caused any significant change in mitochondrial membrane potential at 4 h, but after treatment with TW80-SeNPs combined with metformin, the JC-1 value increased to 25.79% compared with 0.19% in the control group. After 8 h, JC-1 increased slightly in the TW80-SeNPs group (4.58%), although there was no significant change in the metformin group. In the combined treatment group, the value of JC-1 increased significantly to 87.98%. After 12 h, compared with the control group (0.19%), the JC-1 value in the TW80-SeNPs group increased to 20.04%, and the JC-1 value in the metformin group increased to 14.15%. In the combined treatment group, its value soared to 95.97%. These indicate that the effect of treatment on JC-1 is time dependent, that is, the value of JC-1 increases with treatment time. The change in JC-1 was more obvious after treatment with the combination of TW80-SeNPs and metformin. Therefore, this combination can more effectively reverse mitochondrial membrane potential and cause mitochondrial damage owing to its synergistic effect.

A change in mitochondrial membrane potential will affect the morphology of the mitochondrion. The morphological changes of mitochondria were observed under a 100 × oil microscope. As shown in Figure 5D, the morphology of mitochondria did not change significantly after 2–4 h, and the outer filaments were intact and did not show obvious breakage. However, at 8–12 h, strand breakage of filamentous mitochondria was observed. This further demonstrated that the combination of metformin and TW80-SeNPs could increase ROS levels in MCF-7 cells, reverse mitochondrial membrane potential, and cause mitochondrial damage.

Selenoproteins are key biomolecules required for the physiological functions of selenium, which include antioxidant activity and metabolic regulation (Hariharan and Dharmaraj, 2020). To determine whether the regulation of selenoproteins was influenced by SeNPs or by the increase in intracellular ROS levels after combined drug treatment, we measured the mRNA expression levels of several selenoenzymes related to the regulation of redox balance. Deiodinase (Dio) is a selenium-dependent enzyme that catalyzes the transformation of thyroid hormone from T4 to T3 (Marsan and Bayse, 2020). The levels of thyroid hormones affect the synthesis of selenoproteins in tissues throughout the body, and Dio indirectly affects the immune response. Glutathione peroxidase (GPx) protects biomolecules against oxidative stress. (Adriani et al., 2021). As shown in Figure 5E, the relative expression of Dio1/2 decreased to a certain extent after TW80-SeNP treatment, the expression of GPx2/4/6 also decreased, and the expression of SelR decreased slightly. This results suggest that, under the effective concentrations, TW80-SeNP could decrease selenoprotein levels by inhibiting Dio1/2 expression while simultaneously downregulating the expression of GPx2/4/6 and SelR. Thioredoxin reductase (TrxR) plays a key part in cell growth and proliferation by reducing nucleotides involved in DNA synthesis and maintaining the redox state in cells (Nj et al., 2020). The expression levels of TrxR1/2/3 were slightly increased, as shown in Figure 5E. As shown in Figure 5F, TW80-SeNPs had the least effect on oxidative-stress-related selenoproteins among the various functional SeNP samples studied. Therefore, under the effective concentrations, TW80-SeNPs may indirectly decrease selenoprotein levels by inhibiting Dio1/2 expression while simultaneously downregulating the expression of GPx2/4/6 and SelR, leading to an increase in the oxidative stress response and ROS levels.

### TW80-SeNPs Combined With Metformin Inhibit Cell Growth Through Cell Cycle Arrest

**FIGURE 6:**
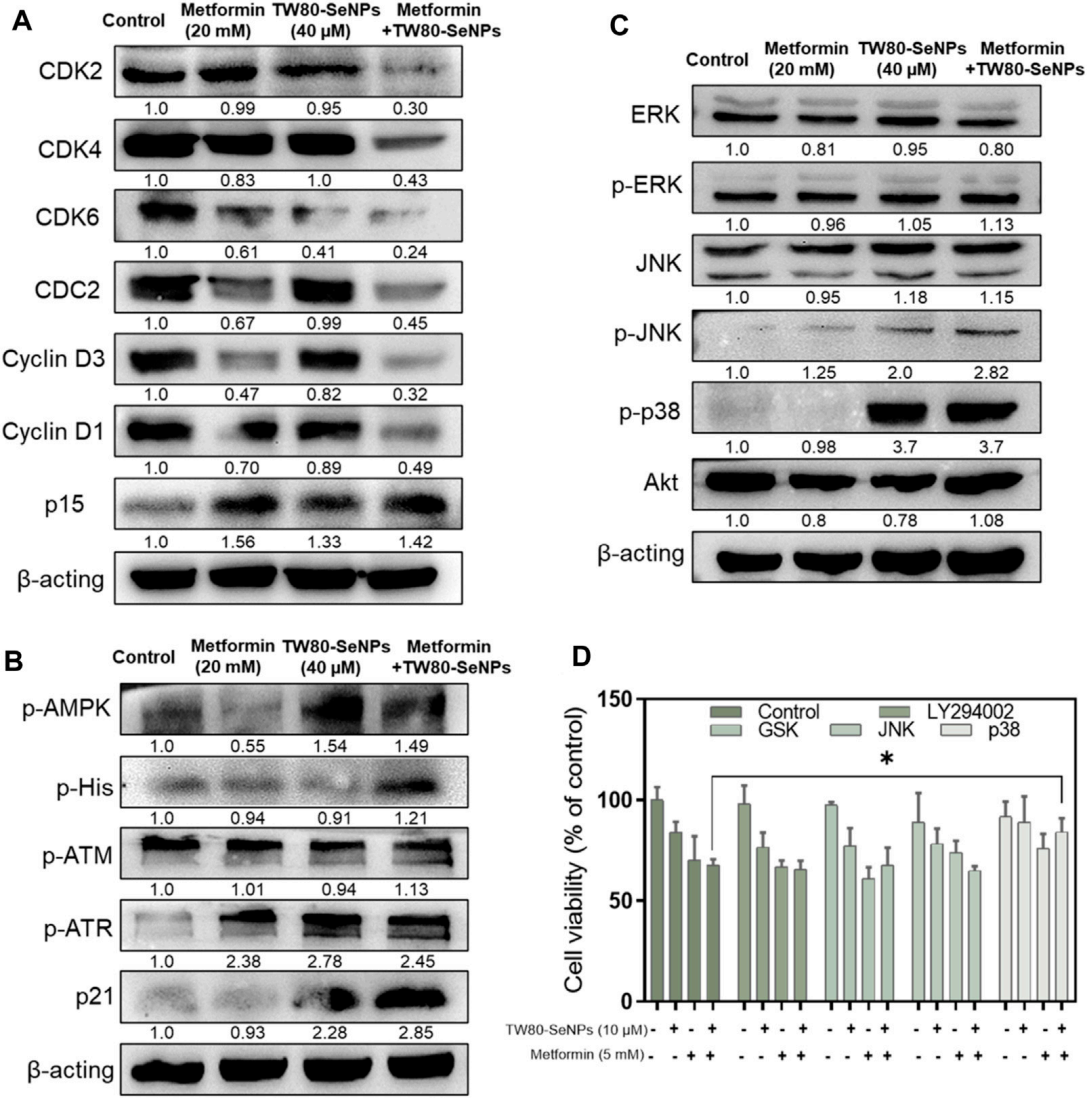
Effects of TW80-SeNPs combined with metformin on expression of signaling proteins in cells. **(A)** Proteins associated with cell cycle arrest. **(B)** Proteins related to DNA damage. **(C)** MAPK-related proteins. **(D)** Cell survival rates after addition of signaling protein inhibitors.

Cyclins and cyclin-dependent kinases (CDKs) have important roles in cell cycle regulation (Saeed et al., 2015; Ding et al., 2020; Sundar et al., 2021). Altering their expression can disrupt the cell cycle. As described above, flow cytometry analysis showed that after MCF-7 cells had been treated with TW80-SeNPs and metformin, the proportion of cells in S phase increased significantly. Western blotting was used to detect the expression levels of CDKs and cyclins in cells treated with the two drugs and with the two-drug combination. As shown in Figure 6A, CDK2/4/6 expression was downregulated after TW80-SeNPs or metformin treatment, and was further significantly decreased when TW80-SeNPs and metformin were combined. Cyclin D1/D3 expression also showed a downward trend after drug treatment, especially after the combination of the two drugs. Moreover, the expression of p15 was upregulated after the combined treatment, suggesting that this treatment affects the regulation of the cell cycle *via* effects on CDKs, cyclin-related proteins, and p15. p21 is a cyclin-dependent kinase inhibitor, which can regulate the cell cycle and DNA synthesis, and thus inhibit the growth of tumor cells. Therefore, we studied the expression levels of p21. As shown in Figure 6B, expression levels of p21 increased significantly after treatment with TW80-SeNPs or metformin, and the effect of TW80-SeNPs combined with metformin was even more significant. Excessive production of ROS can promote the expression of p21 by causing DNA damage and corresponding protein changes. Therefore, we studied the expression of DNA-damage-related proteins p-ATM and p-ATR. The expression levels of these proteins increased after treatment with TW80-SeNPs and with metformin, and increased further after combined treatment, indicating that DNA damage plays an important role in the synergistic effect of TW80-SeNPs and metformin. Metformin inhibits cell growth by affecting the AMPK signaling pathway. Therefore, we studied the expression of p-AMPK in cells. As shown in Figure 6B, we found that the combination of TW80-SeNPs with metformin could promote the expression of p-AMPK, indicating that AMPK has an important role in the synergism of the two-drug combination.

The mitogen-activated protein kinase (MAPK) and Akt signaling pathways play important parts in cell growth, development, differentiation, and apoptosis (Shukla and Gupta, 2007; You et al., 2017). The p38, JNK, ERK, and Akt signaling proteins have important roles in these pathways. Their overall expression levels and phosphorylation were studied here. In Figure 6C, the expression of p-p38 in the TW80-SeNPs group and combined treatment group both increased significantly after treatment. There was no significant change in the expression of Akt, ERK and p-ERK, although p-JNK showed a slight upward trend. To further verify the role of these protein signals, we used the corresponding protein inhibitors for MTT detection. As shown in Figure 6D, after the addition of ERK, JNK, and Akt inhibitors, the cell survival rate showed no significant change compared with the rate observed without the addition of inhibitors; by contrast, after the addition of p38 inhibitors, the cell survival rate increased significantly compared with that observed without the addition of inhibitors. These suggest that p38 has an important role in the enhanced efficacy of the combination of drugs.

## Conclusion

Possible synergistic anti-tumor effects of functionalized SeNPs combined with metformin in breast cancer cells were explored, and TW80-SeNPs combined with metformin were found to significantly kill MCF-7 cells. After further study, a signaling pathway diagram of the synergistic anti-tumor effect was proposed. TW80-SeNPs decreased the expression levels of selenase GPx2/4/6 and Dio1/2 in cells, which resulted in increased oxidative stress response and ROS levels. On the one hand, excessive production of ROS causes mitochondrial damage and activation of AMPK signal, affects mitochondrial energy pathway and inhibits the growth of tumor cells. On the other hand, the expression of DNA damage-related proteins p-ATM, p-ATR and p21 was induced, and the expression of proteins related to cell cycle arrest (CDKs and Cyclins) was down-regulated. Metformin also inhibited the expression of cell block related proteins such as CDC2. The combination of the two can further induce cell cycle arrest and have more significant anti-tumor effect. Therefore, functionalized SeNPs combined with metformin have possible applications in the treatment of diabetes complicated by tumors.

## Data Availability

The raw data supporting the conclusion of this article will be made available by the authors, without undue reservation.
