# Supplementary material for: Functionalized Selenium Nanoparticles Synergizes With Metformin to Treat Breast Cancer Cells Through Regulation of Selenoproteins
Source: Front Bioeng Biotechnol. 2021 Oct 4;9:758482. doi: 10.3389/fbioe.2021.758482 (PMC8543061; doi:10.3389/fbioe.2021.758482)
Supplement: Supplementary file 1 [file Table1.DOCX]

Supplementary Material

Functionalized Selenium Nanoparticles Synergizes with Metformin to Treat Breast Cancer Cells through Regulation of Selenoproteins

Yu Yang^†^, Zehang Zhang^†^, Qi Chen, Yuanyuan You, Xiaoling Li, Tianfeng Chen*

^†^These authors have contributed equally to this work and share first authorship

Department of Chemistry, Jinan University, Guangzhou 510632, China;

*** Correspondence:**Tianfeng Chen
Email: [tchentf@jnu.edu.cn](mailto:tchentf@jnu.edu.cn)

# Supplementary Data

**1.1 Materials**

PVP, PAH, TW80, Na_2_SeO_3_, V_c_, propidium iodide (PI), Hoechst 33342, JC-1 dye, mito-tracker Red, 2',7'-dichlorofluorescein diacetate (DCFH-DA), and 4% paraformaldehyde were purchased from Sigma. Triton-X-100 was purchased from Aladdin. BCA protein quantitative kit, sodium dodecyl sulfate polyacrylamide gel electrophoresis (SDS-PAGE) protein loading buffer (5×), and crystal purple were purchased from Biyuntian. Human breast cancer cell lines (MCF-7 and MDA-MB-231) were purchased from the ATCC Global Biological Resources Center in the United States. Total RNA was extracted using TRIzol (Life Technologies, Grand Island, NY, USA) and subjected to reverse transcription using an ReverTra Ace kit (Toyobo, Osaka, Japan). Real-time PCR was performed using a SYBR Green PCR Master Mix kit (Toyobo) and Opticon 2 real-time Cycler instrument (Bio‐Rad, Hercules, CA, USA). The antibodies involved in the experiments were purchased from Cell Signaling Technology.

**1.2. Cell culture**

Cell lines were cultured at 37 °C (5% CO_2_, 95% humidity) in cell medium (DMEM) containing 10% fetal bovine serum, 1% double antibody: 100 units/mL penicillin, and 50 units/mL streptomycin.

**1.3** **Quantitative real-time polymerase chain reaction (PCR)**

Total RNA was extracted from the cells using TRIzol and reverse transcription was performed using the ReverTra Ace kit. The SYBR Green PCR Master Mix kit and Opticon 2 real-time Cycler were used for real-time PCR. Quantitative PCR was performed using a two-step cycle regimen, starting with a 30-s hot start at 95 °C, followed by 40 cycles at 95 °C for 5 s and 60 °C for 30 s. The primer sequences of selenoproteins were described in Supplementary **Table S1**.

- 1. **Western blot**

MCF-7 cells in logarithmic growth phase (8×10^5^ cells/ mL) were inoculated into a 10 cm cell culture dish, adhered to the wall for 24 h, and drugs TW80-SeNPs (40 μM) and Metformin (20 mM) were added. After TW80-SeNPs (40 μM) combined with metformin (20 mM) treatment for 48 h, the cell status was observed. Remove the supernatant medium, wash with pre-cooled PBS for 2 times, and blot the residual liquid. RIPA lysate of 100~150 μL/min was added and incubated at 4℃ for 10 min. Cells were collected at 4℃ with a cell scraper. The RIPA cracking liquid samples were collected by ultrasonic vibration with an ultrasonic crusher for 1 min. The sample was centrifuged at 4℃ for 20 min, and supernatant was collected. The concentration of collected protein (3~4 μL supernatant) was detected by BCA protein kit. After the concentration of protein sample is tested, add the protein solution with the same concentration in a certain volume, and mix it evenly with the ratio of protein sample: Loading Buffer (5×) = 4:1. The oscillating mixture was heated at 95℃ for 5 min at 300 rpm by a microporous oscillating heater. Allow the solution to cool to room temperature and then freeze at -20℃ or -80℃ for later use.

SDS-PAGE electrophoresis: Different proportions of separation glue (10%~15%) were prepared according to the molecular weight of protein signal detected. 4.5-5 mL of separation glue was added between each electrophoresis glass plate, and 1 mL of isopropanol was added for leveling the surface of separation glue. After 30-50 minutes of separation glue coagulation, isopropanol was absorbed with filter paper. Do not touch the separation glue, quickly add the prepared 4% concentrated glue and insert it into the comb that has been cleaned and prepared. After the concentrated glue solidifies, put it in a moist plastic bag and store it in the refrigerator at 4℃. The next day, the plate was submerged in a prepared electrophoretic buffer. Pull out the comb quickly and firmly. The prepared protein sample (80 μg/ well) and Marker (8 μL) were added. The electrophoresis conditions were set as follows: voltage 70 V, time 30 min (for running concentrated glue); Voltage 110V, time 75 min (for running separation glue). The electrophoresis conditions of the body should be adjusted appropriately according to the specific situation. When the protein sample is about through the separation glue, the power is stopped. Pry open the rubber plate and cut the rubber block, and prepare for the film transfer. The prepared transfer buffer is placed on a plate and soaked in pre-cooled methanol for several minutes to activate the PVDF membrane. The experimental apparatus was placed in the trans-buffer according to the prescribed order: sponge pad - filter paper -PVDF membrane - adhesive - filter paper - sponge pad, and the bubbles on the contact surface between PVDF membrane and adhesive block were carefully removed, so as not to affect the experimental results. The assembled apparatus was placed in trans-buffer and the membrane was transferred under ice bath conditions. Conditions of film transfer: voltage 110 V, time 90 min. After membrane transfer, PVDF membrane was removed and placed in 5% milk sealant for 2 h at 25℃.

At the end of sealing, the sealing fluid is recovered. The PVDF membrane was washed with 1×TBST buffer solution for 3 times, 10 min each. The prepared diluent of primary antibody (1:1000) was added into PVDF membrane and incubated overnight at 4℃. Next day, the diluent of primary antibody was recovered. The membrane was washed with 1×TBST buffer solution for 3 times, 10 min each. The diluted secondary antibody (mouse or rabbit source, 1:2000) corresponding to the primary antibody was added and incubated at 4℃ for 2 h, then the diluted secondary antibody was recovered. The membrane was washed with 1×TBST buffer solution for 3 times, 10 min each. The PVDF membrane was removed and 100~200 μL of ELC luminescent solution was added (ELC kit VA liquid: VB liquid =1:1). The PVDF membrane was incubated in the dark for 1 to 2 min, and then developed in a chemiluminescence apparatus. The protein expression of the exposed bands was analyzed semi-quantitatively with ImageJ software.

# Supplementary Figures and Tables

## Supplementary Figures


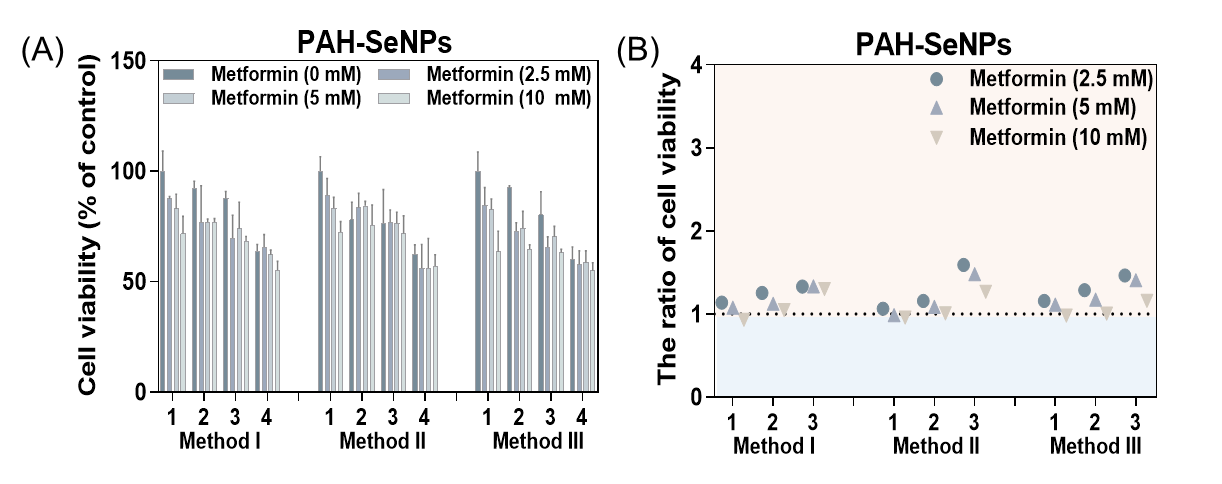


**Supplementary Figure 1.** (A) Effects on MDA-MB-231 cell viability of PAH-SeNPs combined with metformin under three treatment methods, where 1, 2, 3, and 4 represent Se concentrations of 0, 1, 2 and 4 μM, respectively. (B) Survival rates of cells with and without SeNPs and the same concentration of metformin. The horizontal coordinates 1, 2 and 3 represent Se concentrations of 1, 2 and 4 μM, respectively. Points greater than 1 on the ordinate indicate some synergy.

**
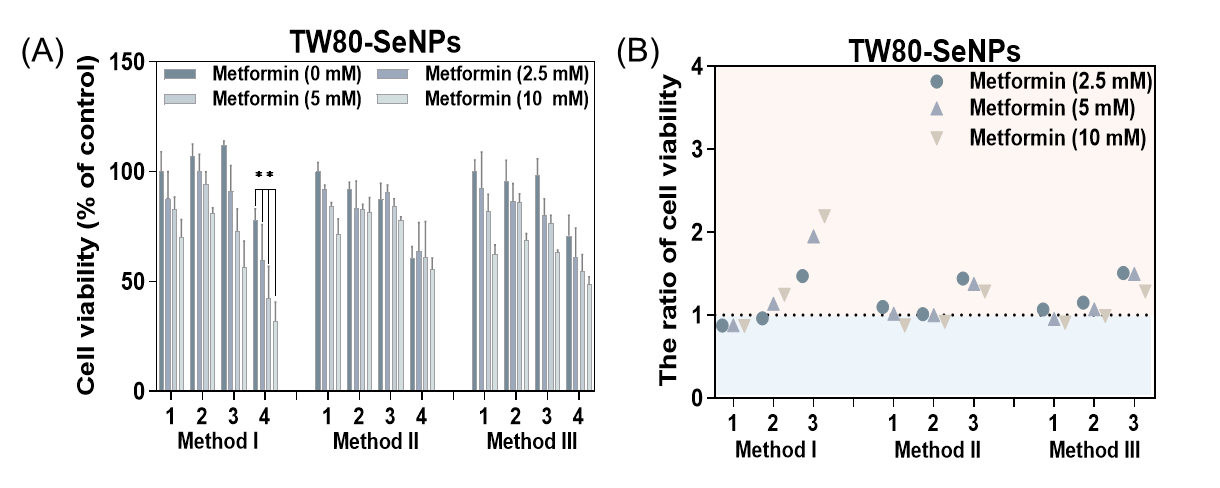
**

**Supplementary Figure 2.** (A) Effects on MDA-MB-231 cell viability of TW80-SeNPs combined with metformin under three treatment methods, where 1, 2, 3, and 4 represent Se concentrations of 0, 1, 2 and 4 μM, respectively.  ** *P* < 0.01, with significant statistical difference. (B) Survival rates of cells with and without SeNPs and the same concentration of metformin. The horizontal coordinates 1, 2 and 3 represent Se concentrations of 1, 2 and 4 μM, respectively. Points greater than 1 on the ordinate indicate some synergy.


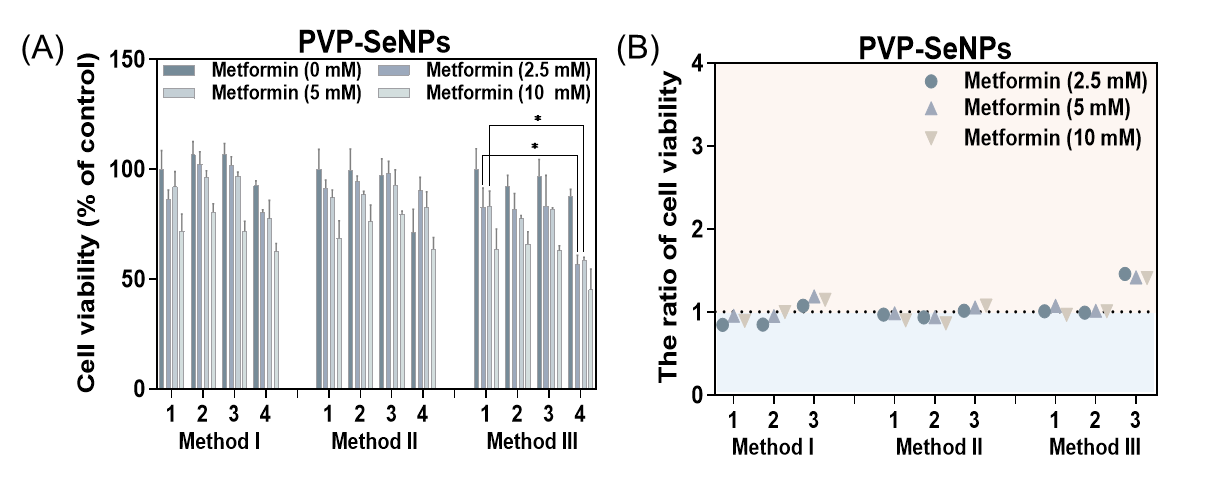


**Supplementary Figure 3.** (A) Effects on MDA-MB-231 cell viability of PVP-SeNPs combined with metformin under three treatment methods, where 1, 2, 3, and 4 represent Se concentrations of 0, 1, 2 and 4 μM, respectively. * *P* < 0.05, with significant statistical difference. (B) Survival rates of cells with and without SeNPs and the same concentration of metformin. The horizontal coordinates 1, 2 and 3 represent Se concentrations of 1, 2 and 4 μM, respectively. Points greater than 1 on the ordinate indicate some synergy.

## Supplementary Tables

**Supplementary Table 1**. Selenoproteins and their primer sequences

| Selenoproteins | The primer sequences |
| --- | --- |
| Dio1 (Forward)  Dio1 (Reverse)  Dio2 (Forward)  Dio2 (Reverse)  Dio3 (Forward)  Dio3 (Reverse)  GPx1 (Forward)  GPx1 (Reverse)  GPx2 (Forward)  GPx2 (Reverse)  GPx3 (Forward)  GPx3 (Reverse)  GPx4 (Forward)  GPx4 (Reverse)  GPx6 (Forward)  GPx6 (Reverse)  SelR (Forward)  SelR (Reverse)  TrxR1 (Forward)  TrxR1 (Reverse)  TrxR2 (Forward)  TrxR2 (Reverse)  TrxR3 (Forward)  TrxR3 (Reverse) | TTATGCAAGGTAATAGGCCA  TCTTAAAAGCCCAGCCATC  TGGACAATAACGCCAACATA  CGGACTTCTTGAAGGTTGTA  CTCTGGCTTCTCGATTTCTT  CCTGCTTGAAGAAATCCAAC  TCGGTGTATGCCTTCTCGG  CGTTCTCCTGATGCCCAAA  GATGACCCATTTTCCCTCAT  ATGTCAGGCTCAATGTTGAT  CCATTCGGTCTGGTCATT  CCTGGTCGGACATACTTG  AGGGAGTAACGAAGAGATCA  GATGAGGAACTTGGTGAAGT  CATGATATCCGCTGGAACTT  TGAACTGCTTTAGGTACTCC  CGTTCACCGAGACCATTC  GCCTTTAGGGACAAACTTCA  GGGACAGAATGATAGAAGC  CTAAACCAATACCAGCAAG  TGGACTACGTGGAACCTTCTC  CGCCTGGTGCATCAGCT  AAAACATTAGTGGTGGGTG  GGAGAAGGATTGAGCGTA |
